# Membrane-Associated Flavivirus Replication Complex—Its Organization and Regulation

**DOI:** 10.3390/v13061060

**Published:** 2021-06-03

**Authors:** Eiji Morita, Youichi Suzuki

**Affiliations:** 1 Department of Biochemistry and Molecular Biology, Faculty of Agriculture and Life Science, Hirosaki University, 3 Bunkyo-cho, Hirosaki-shi 036-8561, Japan; 2 Department of Microbiology and Infection Control, Faculty of Medicine, Osaka Medical and Pharmaceutical University, 2-7 Daigaku-machi, Takatsuki 569-8686, Japan

**Keywords:** flavivirus, replication complex, replication organelle, endoplasmic reticulum, membranous microenvironment, cellular factors, interferon-stimulated genes

## Abstract

Flavivirus consists of a large number of arthropod-borne viruses, many of which cause life-threatening diseases in humans. A characteristic feature of flavivirus infection is to induce the rearrangement of intracellular membrane structure in the cytoplasm. This unique membranous structure called replication organelle is considered as a microenvironment that provides factors required for the activity of the flaviviral replication complex. The replication organelle serves as a place to coordinate viral RNA amplification, protein translation, and virion assembly and also to protect the viral replication complex from the cellular immune defense system. In this review, we summarize the current understanding of how the formation and function of membrane-associated flaviviral replication organelle are regulated by cellular factors.

## 1. Introduction

The genus Flavivirus of the Flaviviridae family is a large virus group comprised of many emerging arthropod-borne pathogens. Flaviviruses are predominantly transmitted by mosquitoes and ticks and include clinically important viruses: Japanese encephalitis virus (JEV), dengue virus (DENV), yellow fever virus (YFV), West Nile virus (WNV), and tick-borne encephalitis virus (TBEV), which cause life-threatening diseases such as hemorrhagic fever, encephalitis, and meningitis in humans [1]. Furthermore, the recent explosive outbreak of Zika virus (ZIKV), mainly in South and Central America, has revealed that infection with this flavivirus is responsible for congenital microcephaly [2]. Therefore, flavivirus infections are continuing global threats to public health. However, there are no specific antivirals available for most flavivirus infections.

Flavivirus is a lipid-enveloped RNA virus. Its ~11,000-nucleotide-long single-stranded positive-sense RNA (ssRNA(+)) viral genome contains a single long open reading frame (ORF) encoding three structural (capsid [C], pre-membrane [prM], and envelope [E]) and seven non-structural (NS1, NS2A, NS2B, NS3, NS4A, NS4B, and NS5) proteins, flanked by highly structured 5′ and 3′ untranslated regions (UTRs). Structural proteins are required for the formation of infectious viral particles, whereas NS proteins play important roles in viral RNA replication, protein processing, and virion assembly [1]. Additionally, some of the flaviviral NS proteins have been reported to counteract the interferon (IFN)-mediated antiviral response in virus-infected cells [3]. 

Flavivirus infection begins with the binding of E glycoproteins with cell-surface entry receptors. A number of cellular molecules have been shown to be entry receptors for flavivirus infection, including heparan sulfate, C-type lectins, and phosphatidylserine receptors [4]. Internalization of the attached virion is mediated by clathrin-dependent endocytosis. Subsequently, a fusion between viral and endosomal membranes occurs, which is triggered by the structural rearrangements of the E protein induced in the acidic environment of the endosomal vesicles. After penetration of the nucleocapsid into the cytoplasm, the single ORF is translated from viral RNA, which is disassociated from C proteins, and a precursor polyprotein is co- and post-translationally cleaved by viral and host-encoded proteases. The viral protein involved in this polyprotein processing step is NS3, which possesses helicase, RNA triphosphatase, and serine protease activities [5,6].

The viral ssRNA(+) also serves as a template for the synthesis of new copies of genomic RNA, in which negative-sense RNA is first generated and then directs the amplification of new positive-sense RNAs. This viral RNA synthesis is catalyzed by the RNA-dependent RNA polymerase (RdRp) activity of NS5. During the viral RNA synthesis process, the N-terminal portion of NS5, which is reported to contain guanylyltransferase (GTPase) and methyltransferase (MTase), involves the formation of a type 1 cap (m7GpppAmp) structure at the 5′ end of the viral RNA [5,7].

This viral RNA amplification process takes place on the endoplasmic reticulum (ER) membrane, where the so-called “replication complex” is formed. Subsequently, the nucleocapsids, composed of C proteins and synthesized ssRNA(+), are assembled with prM and E heterodimers and cellular lipid bilayers to form immature particles in the ER lumen. At this budding step, the prM makes the immature particles non-infectious by repressing the fusogenic activity of the E protein. However, during transport through the secretory pathway, the conformational change of glycoproteins takes place in the acidic environment of the trans-Golgi network (TGN), resulting in processing of the prM by the cellular protease, and thereby creating a mature virion [8,9]. Then, the infectious virion egress from the infected cell occurs through exocytosis.

The process of the replication complex formation requires many viral and cellular factors [10]. For instance, some trans-membrane NS proteins function as the scaffold to retain the replication complex to ER. Among them, NS2B serves as a cofactor of NS3 protease activity [5]. Additionally, DNAJC14, a cellular protein chaperone, is shown to facilitate the viral protein processing step, resulting in the formation of a functional replication complex [10,11]. Of particular interest is that the intracellular membrane structure within the ER is rearranged by a virus infection, which is closely associated with the activity of the flaviviral replication complex, including viral RNA amplification and protein translation [12,13,14,15,16,17,18,19,20]. The establishment of this membranous microenvironment is thought to be necessary to promote flavivirus replication. Additionally, this architecture wrapped by the ER membrane is presumed to contribute to the protection of the viral replication machinery from cellular immune defense systems [21,22,23,24]. In this review, we will focus our attention on the biogenesis and regulation of the flavivirus-induced organelle-like membranous structures by viral and cellular factors.

## 2. Flavivirus Infection Rearranges Intracellular Membrane System and Creates Organelle-Like Structures

**Figure 1:**
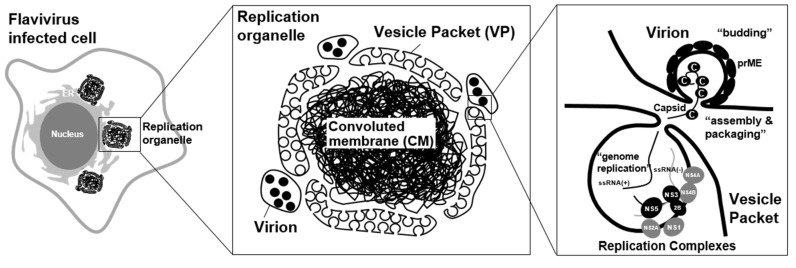
Flavivirus replication organelle. Virus antigen-positive structure was observed in flavivirus infected cells. These structures, called viral replication organelle, are composed of two distinct compartments: a vesicle packet (VP) and a convoluted membrane (CM). VP contains a few dozen small membrane vesicles, and the inner areas of these vesicles are connected to the cytosol through small pores. VPs have been considered to be the location of viral genome replication. The progeny positive-sense RNAs are synthesized in the VP vesicles by the viral replication complexes. The newly synthesized genomic RNAs are exported to the outside of the vesicles to be assembled into infectious particles at a proximal area of the vesicle pore [28]. A CM is forming a reticulovesicular network of membranes. Since it contains abundant viral non-structural membrane proteins, the role of the CM was previously proposed to be as the location for the processing or storage of viral polyproteins [8].

Flaviviruses induce a unique organelle-like structure, called a “viral replication organelle,” in the cytoplasm of infected cells [25,26]. Since the viral proteins and double-strand RNA (dsRNA) are detected within the viral replication organelle [26], this could be the location of viral genome replication, viral protein synthesis, and viral particle assembly. Although the size of this structure varies in each flavivirus species and host cell type, several ER marker proteins have been detected in this structure, suggesting that the membrane of the viral replication organelle is derived from the ER in all cases. The imaging analysis using probes for high-mannose glycans, such as concanavalin A (CoA) and wheat germ agglutinin (WGA), revealed that the structure could originate from the pre-Golgi area of the ER [15]. The viral replication organelle has been considered to be composed of two distinct compartments: a vesicle packet (VP) and a convoluted membrane (CM) (Figure 1). 

Several electron tomography analyses of flavivirus-infected cells showed that a VP approximately 50–90 nm in diameter and containing a few dozen small membrane vesicles appears [27]. Further, 3D imaging analysis revealed that the membranes of luminal small vesicles are not separated from the packet membranes, and the inner areas of these vesicles are connected to the cytosol through small pores. Since dsRNA, which is an intermediate product of the viral genome replication, is detected in the VP area of the viral replication organelle via immuno-fluorescent imaging analysis, VPs have been considered to be the location of viral genome replication. Previously, a model was proposed in which the progeny positive-stranded RNAs are synthesized in the VP vesicles by the viral replication complexes, and the newly synthesized genomic RNAs are exported to the outside of the vesicles to be assembled into infectious particles at a proximal area of the vesicle pore (Figure 1) [28].

Unlike a VP structure, a CM is observed as an electron-dense structure in EM analysis, probably forming a reticulovesicular network of membranes. Since it contains abundant viral non-structural membrane proteins, the role of the CM was previously proposed to be as the location for the processing or storage of viral polyproteins [14]. However, its morphology seems to resemble a smooth ER, and no ribosome is detected in the CM, so it is now considered to be a place for lipid storage rather than polyprotein synthesis [14]. Considering that CM formation disrupts the mitochondrion-associated membranes (MAMs), which serve as a platform for MAVS (mitochondrial antiviral signaling)-mediated innate immune signaling, this structure may play a role in the suppression of innate immune signaling [22]. Interestingly, the CM is not always detected in flavivirus-infected cells. For instance, CM structures sometimes could be hardly observed in DENV-infected mosquito cells [29] and ZIKV-infected human neural progenitor cells (hNPCs) [30], but the condition for this structure to appear has not yet been defined. Besides the CM, a zippered ER (zER) membrane, which is a closely aligned ER cisterna, was observed in the ZIKV-infected hepatoma cells [30]; however, the role of this structure is unknown. In TEBV-infected tick cells, the formation of the VP was less abundant compared to infected mammalian cells. Instead, membrane-associated end-closed tubular structures, which were arranged in fascicle-like bundles and reached up 800 nm in length, were often found. Since the tubules were more abundant in the tick cells persistently infected with TBEV, this unique structure may play an essential role in maintaining persistent infection of flaviviruses in arthropod cells [31].

## 3. Viral Proteins Involved in the Formation of a Viral Replication Organelle

The viral replication organelle biogenesis is initiated by the production of viral proteins from the first ssRNA(+) viral genome. The polyprotein of DENV contains proteins NS1 to NS5, which induce the formation of ER membrane invaginations. These induced structures are very similar to the VPs observed in the DENV-infected cells, which indicates that these NS proteins are sufficient to generate a viral replication organelle [32]. Among them, the membrane-anchored NS1, NS2A, NS4A, and NS4B proteins play central roles in the ER shape deformation to create a viral replication organelle [1,33]. The membrane structures observed in the individual protein-expressing cells are different from those observed in the virus-infected cells, suggesting that the cooperation of these proteins and/or host factors is required for the formation of a viral replication organelle [1].

The expression of a polyprotein lacking the NS1 coding region does not induce VP structure formation, which indicates the essential role of NS1 in this process [32]. NS1 is post-translationally modified via N-linked glycosylation and has six invariant intramolecular disulfide bonds to form a dimer in the ER lumen. NS1 consists of three domains, a core “β-ladder” domain, a small “β-roll” dimerization domain, and a “wing” domain [34]. In the NS1 dimer structure, the NS1 dimer directly binds to the ER membrane via the hydrophobic surface of the protrusion structure created by the β-roll and connector subdomain of the wing, and it plays an important role in membrane bending from the inside of the ER luminal space [34]. Bending of the membrane has been considered to be related to the formation of vesicles during VP production [35] and the production of infectious particles [36].

NS2A, NS4A, and NS4B proteins do not constitute virus particles and do not exhibit enzymatic activities. Among them, NS4A and NS4B have been shown to play an essential role on the membrane deformation during viral replication-organelle formation [37]. NS4A consists of three regions, a hydrophilic N-terminal region in the cytoplasm, a hydrophobic region associated with endoplasmic reticulum (ER) membranes (pTMS1–pTMS3), and a C-terminal region named 2K. The 2K region functions as the signal sequence for translocation of the NS4B protein into the lumen and is cleaved off by NS2B-NS3 viral protease [38]. The NS4A N-terminal region plays role in homo-oligomerization and is supposed to be the region interacting with cellular factor, vimentin [39]. On the other hand, NS4B contains five hydrophobic segments. Two N-terminal segments (pTMD1 and pTMD2) are considered to be associated with the ER luminal side of the membrane, and three C-terminal segments (pTMD3–pTMD5) are anchored into the ER membrane as transmembrane regions [40,41]. NS4B also forms a homodimer in virus-infected cells. The expression of both NS4A and NS4B was able to induce an ER membrane rearrangement that was similar to that observed in infected cells. Therefore, these proteins are considered to be the main driving factors for viral replication-organelle formation [38,41,42]. Both proteins have been demonstrated to be able to interact with other viral proteins, including NS3 localized in the cytoplasm and NS1 positioned in the ER lumen to modulate viral replication [43,44,45].

## 4. Host ER Shaping Proteins Are Involved in the Formation of a Viral Replication Organelle

Some host ER shaping proteins, such as reticulon (RTN) and atlastin (ATL) family proteins, are also involved in membrane deformation during viral replication organelle formation. These proteins are highly conserved in eukaryotes with short, hairpin-like transmembrane domains, which are considered to cause membrane deformation by forming a wedge-like structure that is embedded into the ER membrane at the cytoplasmic leaflet of the lipid bilayer. Aktepe et al. reported that RTN 3.1A, one of the RTN family proteins, is required for flavivirus genome replication. The depletion of RTN 3.1A in the ZIKV-infected or WNV-infected cells decreased membrane curvature formation and replication vesicle production in the VPs. On the other hand, RTN3.1A associates with NS4A, and this interaction probably mediates membrane remodeling to promote vesicle formation [46]. 

ATL family proteins are also involved in the formation of a flavivirus replication organelle [1]. These proteins are localized at the ER membrane and are considered to be involved in the fusion of ER tubules to maintain ER mesh networks [47,48]. Neufeldt et al. reported that the depletions of ATL2 and ATL3 impair the production of infectious DENV and ZIKV particles [1]. ATL2 depletion also suppresses the viral genome replication. Furthermore, the vesicles in the infected cells were condensed within a small perinuclear region, and the vesicle shapes were distorted in the ATL2-depleted cells, suggesting that ATL2 may also be required for the formation of replication vesicles in the viral replication organelle. The requirement of the ATL family for viral genome replication was also reported by Monel et al. [49]. They found that ATL3 is recruited to the viral replication organelle along with the viral NS2A and NS2B3 proteins.

## 5. The Endosomal Sorting Complex Required for Transport (ESCRT) Proteins Is Potentially Involved in Membrane Invagination and Fission

The proteome analysis for the JEV-infected cells revealed that host ESCRT proteins were recruited to the viral replication organelle [50]. ESCRT proteins are involved in membrane invagination toward the luminal side from the cytoplasm and in membrane sealing from the cytoplasmic phase [51]. In the flavivirus-infected cells, depletion of the CMHP2 or CHMP4 family proteins significantly impairs infectious particle production, suggesting that ESCRT-mediated membrane sealing is mainly involved in the viral budding process, rather than VP formation [50]. However, studies using other ssRNA(+) viruses that can infect *Saccharomyces cerevisiae* have suggested a different model. Brome mosaic virus (BMV) and tomato bushy stunt virus (TBSV) infection have been shown to remodel ER membrane and create spherules [52]. This structure is similar to the vesicles in VPs observed in Flavivirus infected cells, because the requirement of host factors, such as RTN family proteins, is the same to that for VPs formation [53]. In a study using BMV and BSV, the genetic analysis of yeast for non-essential genes has clarified that several ESCRT-encoding genes are required for viral RNA genome replication. Furthermore, a lack of ESCRT factors affects the formation of spherules [54,55], suggesting that ESCRT factors play important roles in the viral replication organelle. Studies of flavivirus infection by Tabata et al. also indicated that the depletion of ESCRT factors slightly affects virus genome replication, suggesting that the roles of ESCRT factors may commonly be involved in the VP formation of ssRNA(+) virus replication organelles. 

## 6. Cellular Autophagic Machinery Is Activated in the Flavivirus-Infected Cells

Autophagy is a major bulk-degradation process that involves sequestering the cytoplasmic target with the isolation membrane and delivering its contents to the lysosome. A series of autophagy-related gene (ATG) proteins is known to be required for the autophagosome formations [56]. Autophagy has been identified as a process that supplies nutrients by degrading the cells under starvation conditions, but it is now understood as an important process for the elimination of unnecessary cellular components, such as damaged organelles, protein aggregates, and pathogens [56]. This process is known as “selective autophagy”.

JEV [57], WNV [58], DENV [59], and ZIKV [60] infections activate the autophagic machinery. The accumulation of LC3-II has been observed in these flavivirus-infected cells [61,62]. Pharmacologic inhibition of PI3 kinases associated with autophagy suppresses flavivirus propagation [63]. Furthermore, autophagy activation by treatment with an mTOR inhibitor, such as Torin1 or rapamycin, subsequently increases ZIKV propagation [60,61,64,65], suggesting that autophagic machinery positively regulates flavivirus propagation in the cells. Several indicators, such as that the autophagic isolation membrane could be derived from the ER membrane and some viral proteins co-localize with LC3-II expressed on the isolation membrane, led to the hypothesis that the virus induces autophagy to utilize the autophagic isolation membrane for the materials needed for a viral replication organelle. Recently, Evans et al. have reported that BPIFB3 (BPI fold-containing family B member 3), a regulator of autophagy, is involved in the formation of DENV and ZIKV replication organelles [66]. In this report, the author proposed the model that BPIFB3 has a role in impairing the ER-phagy, selective autophagic degradation of ER, which may negatively regulate viral replication. So far, many different conclusions have been reported; however, the involvement of autophagic machinery in the formation of a flavivirus replication organelle remains controversial [67,68].

Mertz et al., reported that the change of autophagy status after the DENV infection by image-based flow cytometry approach. Interestingly, early phase of infection, basal and activated autophagic flux was enhanced, but in the late stage of infection, autophagosome formation was blocked, and autophagic-degradation capacity was reduced. These results suggest that the requirement for autophagy is different in each replication step [62].

Recently, genome-wide CRISPR-Cas9 screening has identified TMEM41B (trans-membrane protein 41B) as a novel host factor required for flavivirus replication [69]. This protein is involved in the VP formation to protect against the host innate immune response and the induction of apoptosis. TMEM41B has been shown to have roles in the early step of autophagosome formation [70]. Interestingly, the role of TMEM41B in flavivirus replication is independent of autophagy because the phenotype of TMEM41B knockout cells is different from that of Beclin1, ATG5, or ATG7 knockout cells [69]. Its phospholipid scramblases activity, rather than autophagic machinery, may be involved in the viral replication organelle formation [71].

## 7. Flavivirus Infection Changes the Intracellular Membrane Lipid Composition

WNV and ZIKV infection are known to affect the intracellular membrane lipid composition [34,72]. These changes in the membrane lipid composition potentially influence the curvature and thickness of the local membranes [35]. The size balance between the head group and hydrophobic tails affects the shape of the lipid and the spontaneous curvature of the membrane. Specifically, phosphatidylcholine (PC), sphingolipid, and ceramide are enriched in the ER compartments around the flavivirus replication site. The yeast genetics analyses identified choline requiring 2 (Cho2p), a PC synthase, as a host factor required for ssRNA(+) virus replication [36]. PC enrichment in the viral replication organelle is associated with the recruitment of Cho2p by interacting with the viral protein. Treatment with nSMase2 (neutral sphingomyelinase 2) inhibitors, such as GW4869, spiroepoxide, or glutathione, or siRNA depletion of nSMase2 decreases the production of flavivirus subviral particles and leads to the accumulation of viral structural proteins in the cells, indicating that nSMase is required for viral particle secretion [72]. Enzymes involved in the major phospholipid metabolism may play an important role in viral replication organelle biogenesis.

Phosphatidylinositol is also a key molecule in viral replication organelle formation. siRNA knockdown screening identified phosphatidylinositol-4-phosphate (PI4P) kinase (PI4K) as the host factor required for virus replication [73]. PI4P, a product of PI4K, is present in the ER and Golgi apparatus, as well as the viral replication site of other ssRNA(+) viruses, such as picornaviruses [74]. The production of PI4P at the viral replication site can serve as a signal to recruit proteins that have a PH domain, such as the oxysterol-binding protein (OSBP) [75].

Cholesterol is also involved in flavivirus replication. Treatment with a reagent that alters the distribution of membrane cholesterol reduces viral genome replication [76]. The distribution of fatty acid synthases (FASNs) and 3-hydroxy-3-methylglutaryl-coenzyme A reductases (HMGCRs), cholesterol-synthesizing enzymes involved in the production of the cholesterol precursor, is correlated to flaviviral NS proteins [77]. DENV NS3 directly interacts with the FASN and plays a role in recruiting the FASN to the virus replication site. This recruitment facilitates the upregulation of fatty acid synthesis at the viral replication site [78]. HMGCR activities are also increased at viral replication sites. These events affect the local membrane phospholipid composition and are required for the formation of a viral replication organelle [77].

## 8. Cellular Restriction of the Membrane-Associated Flaviviral Replication Complex

As described above, flaviviruses commandeer host biological processes to arrange intracellular membrane systems for efficient viral RNA synthesis, protein production, and particle assembly. Furthermore, it has been demonstrated that cellular factors also act to limit the function of the membrane-associated flaviviral replication complex [10,79]. In particular, the IFN response is crucial in the cellular restriction of flavivirus replication [80].

RNA viruses, including flavivirus, are potent inducers of the IFN-mediated immune response [81]. Upon virus infection, the components of invading viruses such as viral proteins and nucleic acids, which have pathogen-associated molecular patterns (PAMPs), are recognized by host cell-encoded pattern recognition receptors (PRRs) that are primarily found in the extracellular milieu, endosome, or cytosol [82]. As for flavivirus infection, membrane-bound Toll-like receptors (TLR3 and TLR7) and cytoplasmic receptors (retinoic acid-inducible gene I [RIG-I] and melanoma differentiation-associated gene 5 [MDA5]) have been shown to be PRRs detecting viral genomic RNA [83,84,85,86,87]. The recognition of PAMPs by PRRs, in turn, activates downstream signaling cascades, leading to the induction of the production of IFNs and pro-inflammatory cytokines. During the induction of IFN and cytokine productions, stimulator of interferon genes (STING) serves as an adaptor that mediates the signaling of viral RNA detection to the nucleus via interaction with RIG-I. Although STING is ER-associated molecule, activation of the RIG-I signaling pathway induces the translocation of STING to perinuclear puncta, which eventually leads to the phosphorylation and nuclear translocation of IFN regulatory factor 3 (IRF-3) to activate the IFN production. Hence, STING is critical for dsRNA-triggered antiviral innate immunity [88]. Interestingly, a recent study revealed that STING also functions to induce ER-phagy, thereby protecting the host against Gram-positive bacterial infection in mice [89]. On the other hand, DENV is reported to counteract the STING-mediated cellular innate immunity through cleavage of STING by viral NS2B3 protease activity [90,91], suggesting that flaviviruses have evolved their NS proteins to avoid the host immune response [92].

Among three IFN families, type I IFN, the largest IFN family, including IFNα and IFNβ, is considered to play a pivotal role in the innate immune antiviral response; type II (IFNγ) and type III (IFNλ) also induce an antiviral state in cells [80,92]. The IFN produced in an autocrine or paracrine manner binds to the cell surface receptor specific to the respective IFN family and then signals to activate the JAK-STAT pathway through phosphorylation in the cytoplasm. This activation of the JAK-STAT signaling pathway results in the formation of a protein complex that serves as a transcription activator for the expression of hundreds of genes by binding to regulatory elements upstream from the genes on chromosomes [93].

**Figure 2:**
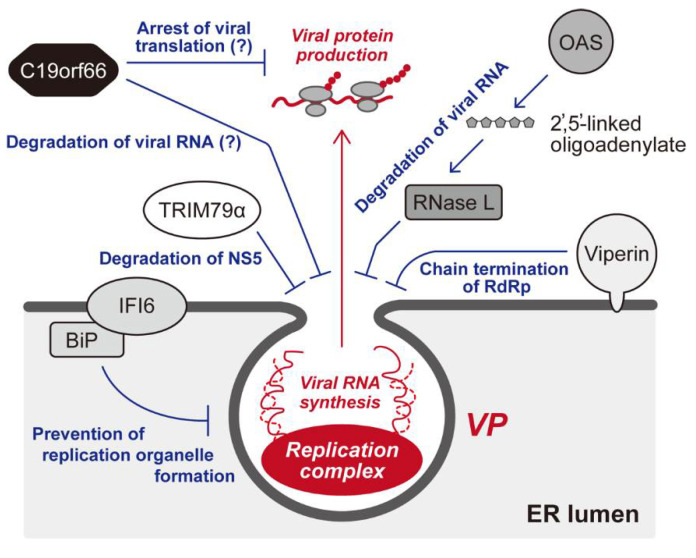
IFN-inducible cellular factors targeting flavivirus replication complex. Several ISGs that are reported to inhibit the activity of membrane-associated replication complex are shown. Recognition of virus-specific dsRNA by OAS produces 2′,5′-linked oligoadenylate, resulting in the activation of RNase L, which in turn degrades viral RNA. Viperin, an ER-associated antiviral ISG, catalyzes the synthesis of ddCTP, which is likely to act as a chain terminator for the flaviviral NS5 RdRp. IFI6 is also shown to be associated with ER, and this ISG is reported to prevent the formation of a replication organelle via interaction with BiP. TBEV replication is shown to be inhibited by TRIM79α, which appears to promote the lysosomal degradation of NS5. C19orf66 (aka RyDEN, IRAV, or Shiftless) is a recently identified ISG that is capable of suppressing the replication of various types of RNA and DNA viruses. As for DENV infection, two modes of inhibition of C19orf66 have been proposed: translational suppression and/or degradation of viral RNA.

The genes induced by the IFN response are called IFN-stimulated genes (ISGs). The IFN has been demonstrated to regulate around 10% of the genome, and nearly 400 genes have been recognized as ISGs whose expressions, to a greater or lesser degree, are upregulated by the IFN response in human cells [80,94]. Importantly, it has become apparent that the products of ISGs include many antiviral factors, which interfere with various steps in DNA and RNA viruses [93]. Not surprisingly, flavivirus infection, which is no exception to this host–pathogen relationship, is also inhibited by antiviral ISGs in some way [79]. The following are ISGs reported to be likely to target the activities of flavivirus replication complexes (Figure 2).

The oligoadenylate synthase (OAS) is a well-studied ISG that collaborates with another IFN-responsive cellular factor, ribonuclease (RNase) L. The OAS is activated by the recognition of dsRNA, a characteristic RNA intermediate structure produced during RNA virus replication, which in turn catalyzes 2′,5′-linked oligoadenylate synthesis from ATP. Subsequently, 2′,5′-linked oligoadenylate activates RNase, resulting in the intracellular degradation of viral genomes [95]. The IFN-induced OAS–RNase pathway has been demonstrated to limit many types of virus infections [96], and flavivirus replications are also inhibited by the expression of OAS and RNase L [97,98,99].

Viperin (virus-inhibitory protein, endoplasmic reticulum-associated, interferon-inducible) is also a well-known cellular inhibitor induced by the IFN against a wide range of viruses, including DENV, WNV, TBEV, and ZIKV [79,100,101]. This ISG is highly conserved among animal species [102], and intriguingly, a viperin-like ortholog was found in the fungal, bacterial, and archaebacterial genes [103]. Although viperin’s antiviral activity has been known for some time, its molecular function was only recently elucidated. Viperin, which contains a radical S-adenosyl-l-methionine (SAM) domain in the central part of the protein, was found to be an enzyme that converts cytidine triphosphate (CTP) into 3′-deoxy-3′,4′-didehydrocytidine triphosphate (ddhCTP) [104]. Accordingly, ddhCTP was shown to function as a chain terminator for the RdRp activity of the RNA virus [104]. Importantly, viperin was shown to be associated with the ER membrane [101] and also co-localized with the complex containing DENV RNA and NS3, suggesting that the primary target of viperin is the flaviviral replication complex [105].

IFNα-inducible protein 6 (IFI6, aka IFI-6-16, or GIP3) is a member of a conserved protein family that consists of IFI6, IFI27 (ISG12a), IFI27L1 (ISG12c), and IFI27L2 (ISG12b) [106], and it was first characterized as a cellular gene induced by the IFN response and virus infections [107,108,109]. IFI6 has been reported to regulate cellular apoptosis [108,110,111]. Although this ISG has been repeatedly identified as a cellular inhibitor against flaviviruses via genetic screening approaches, indicating the important role of IFI6 in the restriction of flavivirus infection [94,112,113,114], the molecular mechanism of the IFI6-mediated antiviral activity had not been sufficiently clarified. However, a recent study has demonstrated that IFI6 localized ER membranes and somehow prevented the formation of a replication organelle in flavivirus-infected cells, resulting in the suppression of efficient viral RNA replication within the membranous microenvironments [115]. In addition, the establishment of an anti-flavivirus state by IFI6 appeared to be assisted by BiP (aka GRP78), an ER-associated chaperone belonging to the heat shock protein 70 family [115]. This evidence suggests that IFI6 acts as an IFN-inducible “guardian” against flaviviruses by preventing the rearrangement of ER membranes.

As demonstrated in studies of other viruses [116], some tripartite motif (TRIM) proteins have been reported to restrict flavivirus replication [79]. The TRIM protein family is characterized by a motif comprised of RING-finger, B-box, and coiled-coil domains (termed an RBCC motif), and the RING-finger domain is responsible for the E3 ubiquitin ligase activity of many TRIM proteins [116]. A murine TRIM protein designated TRIM79α, whose human ortholog has not been found, was shown to inhibit TBEV replication [117]. The expression of TRIM79α is upregulated by type-I IFN treatment and TBEV infection, and interestingly, the inhibitory activity of TRIM79α is somehow specific: TBEV replication was inhibited, but WNV was not. Importantly, TRIM79α induces the lysosomal degradation of NS5 RdRp through direct interaction with NS5, resulting in the suppression of viral RNA replication in infected cells [117]. Although future work will be required to investigate whether the functional TRIM protein directing the degradation of TBEV NS5 exists in humans, the study by Taylor et al. suggests that the flaviviral replication complex can be targeted by TRIM family proteins.

In recent studies by us and others, C19orf66 was reported as a novel ISG that inhibits DENV, WNV, and ZIKV replication [118,119,120,121]. The ISG initially named RyDEN (Repressor of yield of DENV [118], also referred to as IRAV [119] and Shiftless [122] in subsequent studies) has been identified as a cellular gene exhibiting anti-hepatitis C virus (HCV) activity by Schoggins et al., using an overexpression screening of an ISG library [94]. Importantly, it has been shown that the expression of C19orf66 in cultured cells restricts the replication of various types of RNA and DNA viruses belonging to the Togaviridae (Chikungunya virus [CHIKV], Sindbis virus [SINV]), Herpesviridae (herpes simplex virus type 1 [HSV-1], Kaposi’s sarcoma-associated herpesvirus [KSHV]), Adenoviridae (human adenovirus type 3 [hAd3]), and Retroviridae (human immunodeficiency virus type 1 [HIV-1]) families [118,122,123], indicating that this ISG functions as a broad-ranging inhibitory factor. RyDEN is a 291 amino acid protein whose secondary structure was predicted to contain a nuclear localization signal (NLS) in the middle region and a nuclear export signal (NES) in the C-terminal region. In addition, a characteristic glutamic acid–rich domain is found in the C-terminus [118]. C19orf66 mainly resided in the cytoplasm [118] and colocalized with the NS3 and NS4A of DENV and ZIKV [119,120], suggesting that C19orf66 associates with flaviviral RC. Nevertheless, with regard to the C19orf66-mediated inhibition of flavivirus replication, different mechanisms of action have been proposed. In our study, two cellular proteins, poly(A)-binding protein cytoplasmic 1 (PABPC1) and La motif-related protein 1 (LARP1) were shown to interact with C19orf66 [118]. Since PABPC1 and LARP1 were both demonstrated to stimulate the mRNA translation process [124], C19orf66 may block the translation machinery within the replication organelle via interference with the function of the PABPC1/LARP1 complex [118]. Meanwhile, Balinsky et al. proposed that C19orf66 mediates viral RNA decay in the cytoplasmic processing (P) bodies through interaction with P-body-associated cellular factors [119]. Supporting the hypothesis that C19orf66 induces the dysfunction of flaviviral RNA (i.e., translation failure and/or degradation), C19orf66 has been shown to possess RNA-binding activity [118,119]. On the other hand, a study by Wu et al. suggested that C19orf66 directly targets NS3, resulting in the lysosome-dependent degradation of NS3 [120]. Intriguingly, the inhibition of HIV-1 was shown to be attributable to suppression of the -1 programmed ribosomal frameshifting (PRF) of Gag-coding mRNA, resulting in a premature translation product of the Gag-Pol polyprotein [122]. Since the PRF is not involved in the protein expression process of flavivirus replication, the molecular mechanisms underlying C19orf66’s antiviral activity would be diverse.

## 9. Conclusions

The induction of specialized membranous compartments, where genome amplification, protein translation, and particle assembly take place, is a common feature of a variety of viruses. In this respect, flavivirus is a well-studied virus that generates a unique organelle-like structure in the cytoplasm to facilitate virus propagation. As introduced in this review, many cellular factors have been shown to participate in the formation of the replication organelle. On another front, it is becoming apparent that the membrane-associated flaviviral replication complex can be a target of cellular restriction factors as a critical aspect of the IFN-mediated antiviral response. Therefore, further research into the virus–host relationship in the functional regulation of replication organelles will provide a new clue for the development of anti-flavivirus drugs.

## Data Availability

Not applicable.
